# Nursing students’ perceptions about the use of clinical simulation to teach safe medication administration: a focus group study

**DOI:** 10.1186/s12912-025-03716-3

**Published:** 2025-08-18

**Authors:** Cristina Alfonso-Arias, Mireia Llaurado-Serra, Encarna Rodríguez-Higueras, Blanca Goni-Fuste, Laura Brichs-Masnou, Laia Wennberg-Capellades, Maria Angeles de Juan-Pardo

**Affiliations:** 1 Faculty of Medicine and Health Science, Department of Nursing, Universitat Internacional de Catalunya, Josep Trueta s/n. 08195 Sant Cugat del Vallès, Barcelona, Spain; 2 Nursing Faculty, Clinical and Fundamental Nursing Department, Universitat de Barcelona, Feixa Llarga, s/n, 08907 L’ Hospitalet del Llobregat, Barcelona, Spain

**Keywords:** Healthcare simulation, Nursing students, Medication administration, Undergraduate

## Abstract

**Background:**

Safe medication administration is a core competence that nursing students need to acquire during their training. Clinical simulation facilitates the integration of theoretical and practical knowledge in a safe environment, facilitating engagement and greater confidence. However, it is important to integrate students’ perceptions to optimize their learning experience to achieve the desired learning outcomes. The purpose of this study was to explore nursing students’ perceptions about the use of clinical simulation to teach safe medication administration.

**Methods:**

Descriptive qualitative study through focus groups with nursing students who had participated in simulation-based training on safe medication administration where they had been split into two groups that differed only on the degree of fidelity (low fidelity mannequin or standardized patient). Four focus groups were conducted with 24 nursing students who had participated in simulation-based training on safe medication administration. Group discussions were transcribed and subjected to thematic analysis.

**Results:**

Two themes with four subthemes emerged. Theme (1) Usefulness of the clinical simulation for acquiring competence in safe medication administration; included three subthemes. Students reported that simulation-based training helped them link theory and practice, increasing self-awareness of their medication competence and highlighting the importance of training in safe medication administration. Theme (2) “Elements of simulation design that foster learning”; included one subtheme. Students highlighted that having to work individually heightened their sense of responsibility and enabled them to identify their current strengths and weaknesses. The opportunity to observe classmates was seen as useful for learning from mistakes.

**Conclusions:**

The results of this study support the use of clinical simulation to teach nursing students the process of safe medication administration, although it is important that learning scenarios are adapted to students’ level of experience and competence.

## Background

Medication errors remain an important public health challenge. In response to the World Health Organization’s (WHO) *Medication without harm* initiative [1], increased emphasis has been placed on training for health professionals [2]. Safe medication management is a complex and multidisciplinary process in which nurses play a key role, given their responsibility for drug administration [3]. It is therefore important that nurse education provides opportunities for students to acquire the various elements of medication competence [4]. In this context, the integration of theoretical and practical knowledge is crucial for enabling students to understand the complexity of the tasks they must carry out [5]. The learning environment also plays an important role in the acquisition of these skills [6]. Although nursing students must complete clinical placements, ethical and logistical factors related to patient safety may limit their learning opportunities, and thus it is difficult to ensure that all students acquire the same level of competence [7].

Clinical simulation has proven to be an active and effective way of allowing students to acquire knowledge and skills in a controlled setting, avoiding risks to patient safety [8, 9]. Research suggests that simulation-based training is viewed very positively by students, the perception being that it facilitates engagement in the learning process and results in greater confidence in practice than do other teaching methods [10, 11]. However, more needs to be understood about how students perceive their training in relation to safe medication administration and how simulation-based learning might be optimized to achieve desired learning outcomes [12, 13]. The present study explores the perceptions of nursing students about the use of clinical simulation to teach safe medication administration.

## Materials and methods

### Design

This study followed a descriptive qualitative study design involving the analysis of four focus groups conducted with nursing students who had participated in simulation-based training on safe medication administration. In the simulation, students were divided in two different levels of fidelity: mannequin without feedback and standardized patient.

### Participants

Participants were students in year two of a four-year nursing degree program offered by the International University from Catalonia, Spain. All students who had already received simulation-based training during their degree studies were sent an e-mail by the lead researcher inviting them to take part in the study. Those who expressed an interest in doing so were then contacted individually to coordinate their participation in the focus groups. The number of groups required was not established a priori, rather we continued recruiting students until data saturation was achieved with maximum variation of participants and expecting from five to eight students per focus group including participants from both fidelity levels [14]. The focus groups lasted between 60 and 120 min and took place in the simulation lab of our university, the same space in which students received simulation-based training.

### Clinical simulation

The simulation sessions on safe medication administration in which students had taken part were designed in accordance with the *Healthcare Simulation Standards of Best Practice* of the International Nursing Association of Clinical Simulation and Learning [15]. Accordingly, there were three phases: structured *prebriefing* [16], simulation exercise, and structured *debriefing* based on the PEARLS model [17]. Although none of the students had prior experience with a structured simulation of this kind, all participants had attended clinical skills classes related to medication administration during the first year of their program. Prior to the simulation sessions, the class group also attended a two-hour theory class on patient safety in the context of medication administration, which included revision of dose calculation methods.

The clinical simulation exercise required students to administer an IV drug in the university’s simulation facilities. Each teaching session had a total duration of 6 h, spread across three days in 2-hour blocks. Students were split into two groups that differed according to the degree of fidelity, although both groups conducted the simulation in similar physical spaces. One group simulated with a mannequin without feedback in a low- to medium-fidelity scenario, unable to engage with the mannequin during the simulation. The other group worked with a standardized patient who acted as a real patient in a high-fidelity scenario, allowing for direct interaction between the student and the standardized patient. The fidelity of the scenarios was defined according to the three-dimensional model described by Tun et al. [18]. The content of the clinical cases followed was structured around the four stages of safe medication administration established by the WHO: *prescription*, *dispensing*, *administration*, and *monitoring* [19].

Two academic tutors with extensive experience in clinical simulation led the sessions (5–8 years), who designed the case studies and acted as instructors. Each scenario was carefully designed with the learning goals in mind, taking into account contextual aspects such as the expiry date on drug ampules or correct patient identification.

Participants were divided into groups of up to six students, but worked individually during the simulation. While one student performed the simulation exercise, the others observed from an adjacent room via a video link. During the observation, students completed the MEDISIM, a checklist for assessing competence in safe medication administration [20], thus encouraging active learning.

### Data collection

For consistency, all four focus groups were moderated by the same researcher. Another researcher was present as a non-participant observer and took field notes following the recommendations of Krueger and Casey [21]. Both had received previous training in conducting qualitative research and were supervised by a senior researcher with extensive experience of this methodology. The focus groups were audio-recorded for subsequent transcription, and they took place within six weeks of students’ final simulation session. The moderator used an interview guide to facilitate discussion (Table 1).

**Table 1:** Questions guiding the focus group

Topic	Question	Aim
Introduction to the topic	What do you think about simulation as a way of learning about safe medication administration?	Explore general perceptions of simulation as a teaching tool.
Impact of fidelity of the scenario	What’s your view about working with a mannequin vs. a standardized patient in the simulation?	Compare experiences and learning derived from two scenarios (mannequin/actor).
Difficulties and challenges	What difficulties or challenges did you encounter?	Identify barriers and challenges in the simulation sessions.
Proposals for improvement	What would you change or like to see improved?	Gather suggestions about possible changes or improvements to the design of simulation sessions.
Conclusions and wrap-up	Summary of the main points raised, with option to elaborate.	Synthesize the key ideas discussed and allow students to add any relevant information.

### Data analysis

The focus group transcripts and field notes were examined using thematic analysis, as described by Braun and Clarke [22]. Three researchers independently read the material several times to gain an overview of the content and identify units of meaning. This preliminary analysis was then discussed among all researchers to establish patterns of meaning, which were grouped into categories. The three researchers then collectively examined the relationship between categories and grouped them into themes and sub-themes. This proposal was again discussed with the team as a whole to achieve a consensus interpretation. The themes, sub-themes, and categories were recorded in a table, accompanied by direct quotations to support the interpretation. Data were managed and organized using ATLAS.ti 9.

### Rigor

Rigor and trustworthiness of the analysis was assured by following the guidelines for thematic analysis described by Braun and Clarke [22] and by considering the four criteria established by Lincoln and Guba [23], namely credibility, transferability, dependability, and confirmability. To achieve credibility, three researchers first worked independently and then discussed their interpretation in a series of meetings with the whole team to corroborate the findings and ensure a reflective analysis. Credibility was further ensured by sharing the final interpretation with three of the participating students, who confirmed its veracity. To ensure the transferability and dependability of results, the participants and the context in which data were collected are described in detail. Confirmability is supported by the inclusion of numerous direct quotations from the transcribed material and by a clear description of the procedures followed. Rigor in reporting the research was attained by applying the COREQ checklist [24].

### Ethical considerations

The study was performed in accordance with the Declaration of Helsinki and was approved by the university’s Research Ethics Committee (code INF-2018-05). All participants were informed about the nature of the study and it was made clear that participation was entirely voluntary. No incentives (e.g., course credits) were offered for participating. Informed consent was signed before data collection. National and international regulations for research, such as the Declaration of Helsinki, and for data protection have been followed throughout the study. All data were coded before analysis to preserve students’ anonymity.

## Results

Four focus groups involving a total of 24 students (an average of six per group) were conducted. The majority of participants were women (*n* = 19, 97%), and the mean age was 21.4 years (range 19–32 years**)**. Twelve students took part in a high-fidelity simulation with a standardized patient, while twelve worked with a mannequin (without feedback). Interestingly, the initial analysis revealed notable similarity in the experiences and perceptions of students, irrespective of the degree of fidelity (standardized patient vs. mannequin) of the simulated scenario.

Thematic analysis of transcriptions yielded two main themes: (1) Usefulness of the clinical simulation for acquiring competence in safe medication administration, and (2) Elements of simulation design that foster learning. Table 2 shows the themes, sub-themes, and categories that emerged from the analysis, along with illustrative quotations.

**Table 2:** Themes, sub-themes, and categories identified in the thematic analysis of focus groups, with illustrative quotations

Themes	Sub-themes	Categories	Quotations
Usefulness of the clinical simulation for acquiring competence in safe medication administration	Linking theory and practice	Perceiving the theory-practice gap	*“No*,* of course*,* it’s about knowing what the right thing to do is*,* what you want to do as a nurse. But I often have this dilemma when I compare it with the hospital*,* and often it really has little to do with what they tell you to do [in class at university]*,* but I guess the [hospital] nurse trusts what she’s doing and is relaxed about it […] she just gets on with it*,* but often misses certain things. And then you come here [to the university] and you see the importance of keeping a record*,* of noting the dose*,* of signing off…” (A15PGF3)*
		Preparing for clinical practice by developing competence	*“And yes*,* this is real life… it’s not just the drug calculation*,* it’s about how you act in the situation*,* preparing the medication*,* being aware of the patient’s history*,* allergies…” (A17MGF3)*
	Self-awareness of competence in safe medication administration	Identifying personal strengths and weaknesses in relation to safe medication administration	*“You pay more attention to allergies*,* you say to yourself*,* ‘how could I miss that*,* allergy was marked in red’*,* so you‘re really attentive*,* not just in other cases but also in simulation scenarios*,* you remember that you made a mistake in the simulation lab so you’re more attentive*,* perhaps in relation to the 6 rights or whatever…” (A17MGF3)* *“It helped me to be aware of my strengths and weaknesses*,* what I need to improve.” (A16MGF)*
		Awareness of their responsibility and need for decision making	*“…you’re there in the situation*,* and you have to decide whether to take 4 [ml] of potassium or 10 [ml]*,* you have to make the decision*,* it’s down to you […] you might do it right or wrong*,* and it helps you to be aware of whether you’re doing it right or not*,* to really improve…” (A6MGF1)* * “…and there’s responsibility too*,* because I see that it’s up to me*,* I’m the one who has to do it*,* it’s not like when you’re studying… maybe someone could tell me… but it’s me who has to do it. I can ask for help*,* but above all*,* show responsibility in what I’m doing.” (A7MGF1)*
	Recognition of the importance of training in safe medication administration	Importance of prior theoretical training in dose calculation	*“The classroom sessions*,* on dose calculation*,* they were really good*,* I really enjoyed them and came away happy*,* because you had to deal with so many different units on the same sheet*,* so either you got on top of it or you risked making mistakes*,* and for me personally*,* it was great*,* I liked all that number crunching and then putting it into practice.”(A20MGF4)*
		Perceived need for continued simulation-based training in safe medication administration	*“I would repeat it [the simulation] every year or every semester […] it’s good to go over it*,* so you can take it all on board.” (A22MGF4)*
Elements of simulation design that foster learning	Characteristics of the simulation that facilitated learning	Working individually	*“Calculating the dose*,* I find it a real battle as it’s not my forte. You go in there all nervous*,* because of course*,* you’re going to find yourself in that situation. And so you go in knowing it’s your weak point and thinking that you won’t know how to do it*,* but afterwards*,* you’ve had the chance to see yourself in that situation and you can evaluate yourself*,* you can weigh up what progress you have or haven’t made.” (A17MGF3)*
		Formative learning activity	*“For me*,* it’s a relief knowing that it doesn’t count towards your grades*,* so it’s like*,* ‘everything I take away from this is for the good’*,* if I make 6 mistakes*,* they’ll tell me but it won’t matter. When it goes towards your grades*,* then you feel more nervous. I felt really relaxed telling myself ‘I don’t know where I’ve gone wrong*,* and I’m about to find out’*,* and it went really well… the thing that made me most relaxed was knowing that it was only about learning and didn’t count towards my grades.” (A21PGF4)*
		Stress as a precursor of learning	*“[Entering the simulation scenario…] helps you to work under pressure… in my case*,* I get nervous*,* although not to the point of crying*,* so I don’t know*,* it’s like you find yourself in that situation and then you dig deep to get through it.” (A10MGF2)*
		Value of peer observation	*“We’re watching another student and we can say ‘look*,* she’s done it wrong’*,* but then I know what I mustn’t do*,* and if I hadn’t seen that*,* then I’m sure I’d have done it wrong too.” (A1PGF1)* *“It’s good*,* because you see the mistakes that others make and you can think about whether you’d have done the same or not […] you see that others make the same mistakes that you would make. And that gives you the confidence to say: if I get it wrong*,* then I can learn from that.” (A16MGF3)* *“In my class*,* for example*,* when someone was doing the simulation*,* we had the whiteboard and we were noting down the steps they made […] if they’d made a mistake*,* why they’d made a mistake*,* and your classmates explained it to you […] it was really good*,* because you watched your classmate doing it. But at the same time*,* you’re learning from your classmates…” (A17MGF3)*
		Perceived usefulness of simulation with an actor vs. mannequin	*“I always get really nervous*,* whether it’s an actor or a mannequin*,* but the thing is*,* and I don’t know if it’s just me*,* but when it’s a real person*,* I’m more relaxed*,* everything seems more real. I hear the feedback*,* and even if they don’t say ‘you’re doing a good job’*,* just their way of talking makes me more relaxed… With a mannequin*,* I still have to ask all the questions but you don’t get a reply. I’m all the time thinking about what I’ve said*,* because you don’t get a reply. When you’re with a person*,* then you engage in conversation and it’s easier to remember to ask all the necessary questions.” (A20MGF4)* *“That’s what I liked the most*,* the feedback you got*,* like them saying ‘so what are you going to give me?’ You’re living something real*,* it’s not like with a mannequin*,* when you’re there but you don’t know what to say*,* that’s more difficult […] The whole time I was preparing medication and he was saying ‘what are you going to give me’ or whatever […] And that’s how it is in reality*,* isn’t it? Patients are always interested in what you’re giving them*,* so for me*,* it’s better with an actor.” (A15PGF3)* *“Yes*,* it’s very different*,* because there you are preparing the medication and they’re asking you things*,* and while they’re asking*,* you carry on with what you’re doing and it’s really easy to make a mistake with calculating the dose.” (A14PGF2)*

### Theme 1. Usefulness of clinical simulation for acquiring competence in safe medication administration

The first theme refers to the perceived usefulness of the simulation for acquiring competence in safe medication administration. Here there were three sub-themes: Linking theory and practice; self-awareness of competence; and recognition of the importance of training.

### Simulation helps to link theory and practice

Some students compared their experience of the simulation with their observations of professionals when on clinical placement, drawing attention to an important gap between theory (their classroom learning) and practice. In this respect, simulation was considered useful preparation for real clinical practice, not least as it helped them to appreciate the complexity of safe medication administration, a task that involves much more than simply dose calculation.

### Self-awareness of competence in safe medication administration

Some students felt that the simulation had helped them to identify their strengths and weaknesses in relation to medication management, and to be aware of the progress they were making in their learning. Other said that it had helped them to appreciate the nurse’s responsibility and need for decision making when administering medication.

### Recognition of the importance of training in safe medication administration

Some students considered that prior theoretical training in dose calculation had been useful when performing the simulation exercise, although it was suggested that more classroom hours could be devoted to this topic. Similarly, there was a perceived need for continued simulation-based training in safe medication administration across the degree program, introducing progressively more complex cases.

### Theme 2. Elements of simulation design that foster learning

The second theme relates to the design of the clinical simulation, and here there was just one sub-theme, referring to elements of the simulation that facilitated learning.

### Characteristics of the simulation that facilitated learning

Various characteristics of the simulation-based training were considered by students to have facilitated learning. One related to having to work individually, as would be the case in real clinical practice, as this heightened their sense of responsibility and helped them identify their current strengths and weaknesses. Another important issue for some students was that the simulation activities were purely formative and did not contribute to their grades. This reduced the stress associated with the tasks and meant they were not overly concerned with making mistakes, it was all about learning. Although one student did report having felt stressed during the simulation, she said that it did not leave her feeling blocked as she knew she was in a safe environment.

Being able to observe classmates working was also highlighted as a positive aspect, as it helped students become attuned to possible errors and to reflect and learn from the mistakes of others. Completing the MEDISIM checklist during observations was also viewed by most students as useful for promoting their own learning, as was the opportunity to discuss the actions of the student they were observing with classmates.

Finally, many students expressed a preference for working with a standardized patient (actor) rather than a mannequin, as they felt the interaction with a real person made the experience of preparing and administering the medication more realistic. However, this was not a unanimous view, as some students considered that talking with a standardized patient could distract them from aspects of their task, especially the dose calculation. They also felt that calculating the dose and preparing the drug in the presence of the patient was not a realistic scenario.

## Discussion

The purpose of this study was to explore nursing students’ perceptions of the use of clinical simulation in teaching safe medication administration. The results obtained from analyzing four focus group discussions suggest that this approach can make a significant contribution to students’ learning and awareness of this fundamental competence. In particular, students considered that simulation-based training provided good preparation for real clinical practice, helping them appreciate the responsibility that nurses have when administering medication and drawing attention to the challenges of applying their theoretical knowledge in a real clinical setting. They also commented on elements of the clinical simulation that they felt had promoted their learning.

Regarding the nurses’ role in safe medication management, several students noted that the simulation-based training had made them more aware of how the process involves more than simply correct dose calculation. This reflects the accepted view that safe medication management is a complex and multidimensional process, in which nurses play a key role, given their ultimate responsibility for drug administration [4]. Consistent with the perceptions of our students, other authors have found that clinical simulation can enhance students’ understanding of and ability to perform complex nursing tasks [25, 26]. This awareness of the nurse’s role and the factors that may contribute to adverse events is vital for fostering students’ engagement and capacity for critical reflection on their practice, which is essential for minimizing the risk of errors [27, 28]. In this context, a recent review concluded that poor critical thinking skills among nursing students on clinical placement is one of the main causes of medication errors [29].

The fact that students felt better prepared for clinical practice as a result of the simulation-based training supports the use of this approach, which is the stated aim of enhancing competence within a safe environment for both patients and professionals [30]. The WHO recommends that all nursing students should gain experience in simulated scenarios before working with real patients [31]. Accordingly, universities need to ensure that students have sufficient opportunities to acquire the level of competence required for clinical practice [27], not least because there is a widespread perception of a lack of knowledge and experience among newly qualified nurses [2]. Some studies suggest that only 40% of experienced nurses believe their newly qualified colleagues are sufficiently competent in medication management [32].

Participants in the present study had already completed four weeks of clinical placements, and in this respect, it is interesting to note how some of them highlighted differences between what they had been taught at university and the behavior of professional nurses with whom they had worked. This perception of a theory-practice gap has been noted by other authors [33, 34], and it is considered to be bidirectional, that is to say, it may be that clinical practice does not reflect current scientific evidence [35], or that university teaching is not well aligned with everyday clinical realities [36]. These disparities pose a particular challenge for students, whose lack of experience and professional maturity may make it difficult for them to identify the correct course of action [8]. Because clinical simulation offers students the opportunity to observe and reflect on their actions, most notably in the debriefing stage, it can drive and enhance their clinical judgment skills [37]. It should be noted that our students did not refer explicitly to debriefing as an activity that promoted their learning, although they did mention the positive outcomes resulting from it. This is likely because it was their first experience with structured clinical simulation, and therefore, they were unfamiliar with the stages and terminology associated with this teaching method. Future studies should include students with more experience in simulation-based training to compare whether they make more explicit references to the critical reflection that is central to debriefing.

Another issue that emerged in the focus groups was the perceived need for simulation-based training in safe medication administration to continue throughout the degree program, with more complex scenarios being progressively introduced as students gain more experience. This reflects the findings of previous studies, in which students highlighted the importance of increasing their knowledge and skills in medication management through simulation activities, identifying this as critical to their clinical development [10, 33].

The introduction of continued simulation-based training could be complemented by a longitudinal analysis of their competence in medication administration (both perceived and actual) as they progress through their studies, as suggested by the experts in simulation [38]. The longitudinal qualitative literature in clinical simulation is limited; however, several longitudinal quantitative studies emphasize the importance of continuous monitoring to identify areas for improvement, optimize educational strategies, and assess their impact on clinical practice [39, 40]. Jarvill [40] evaluated students at three different stages throughout their training, finding that, despite progress, only one-third of final-year students demonstrated integrated competence. Additionally, it was observed that students who engaged in repeated simulations showed significant improvements in both their skills and confidence. In addition, using a mixed methods research design would make possible to analyze the real impact on clinical practice as well as to deepen in their perceptions to provide key points for the competence maintenance. Moreover, the study by Alfonso-Arias et al. [20] highlights the value of assessment tools such as MEDISIM, which allow for tracking students’ competence development over time, supporting self-assessment and fostering continuous improvement.

On a different note, in the view of many students, the fact that the simulation-based training was formative and did not count towards their course grades was an important aspect. This approach helps create the perception of a *safe container* [16] and increases the likelihood that students feel able to test their skills without fear of making mistakes. Although one student said she had felt stressed before the simulation, this did not prevent her from performing the tasks as she knew she was in a controlled setting. Other authors have noted that clinical simulation can generate stress in nursing students, especially those with less experience [41]. However, when this stress is experienced in what is ultimately a safe environment, it may contribute to the sense of a stimulating challenge that drives learning [42].

Among those students who worked with a standardized patient, many considered the possibility of receiving direct feedback in real-time to mean that the experience more closely resembled clinical practice. Others, however, felt that the interaction with the patient distracted them from preparing the medication, suggesting that this combination of tasks has the potential to produce cognitive overload in new students [43]. Research has found that interacting with a standardized patient in simulation training can be a positive stimulus for learning [25], although students need to be capable of taking advantage of this rather than feeling overwhelmed by the situation. Our results suggest the need to consider students’ previous experience and level of competence, allowing simulation scenarios to be tailored to their current learning stage [43].

### Limitations

One limitation of the present study is that each individual student worked with either a standardized patient or a mannequin, and thus they could not compare their experience with these different levels of fidelity. Future studies should aim to obtain comparative data by exposing students to both approaches. A further limitation is that all students were enrolled in the same nursing degree program, and hence it is unclear to what extent our findings are generalizable to other contexts.

## Conclusion

The results of this study support the use of clinical simulation to teach nursing students the safe administration of medication. Students considered that the simulation-based training had heightened their awareness of the importance of developing their competence in this respect, drawing attention to the potential gap between theory and practice. It also helped them to identify their current strengths and weaknesses, encouraging critical reflection.

The fact that the simulation training was purely formative was seen by students as important for allowing them to test their skills and learn from mistakes, both their own and those of observed classmates. However, the potential for cognitive overload when preparing and administering medication to a real patient highlights the need to adapt simulation scenarios to students’ current level of experience and competence. It was also suggested that simulation-based training in safe medication administration should be continued throughout the degree program, with progressively more complex cases being introduced as students gain confidence. This could help to ensure that nursing students acquire the competence they will need in clinical practice, thereby contributing positively to patient safety.

## Data Availability

The dataset and analysis supporting the conclusions of this article is available under request to the corresponding author, without undue reservation.
